# Trajectories of Depressive Symptoms in Community-Dwelling Korean Older Adults

**DOI:** 10.1093/geroni/igaa057.553

**Published:** 2020-12-16

**Authors:** Jinhee Shin, Eunhee Cho

**Affiliations:** Yonsei University, Seoul, Republic of Korea

## Abstract

Objectives This study aimed to identify trajectories of depressive symptoms and investigate predictive variables of latent class in Korean community-dwelling older adults. Methods Study participants comprised 2,016 community-dwelling Korean adults aged over 65 years, using data from the Korean Longitudinal Study of Aging (KLoSA) from 2006–2016. The KLoSA, a nationally representative panel survey, has been conducted biannually since 2006. We used latent class growth analysis to identify depressive symptom trajectories. Multinomial logistic regression analysis was conducted to identify predictors of each class of depressive symptoms. Results Five depressive symptom trajectory groups were identified: Class 1, no depressive symptom (13.8%); Class 2, low depressive symptom (32.8%); Class 3, decreasing depressive symptom (10.6%); Class 4, increasing depressive symptoms (24.0%); and Class 5, persistent depressive symptoms (18.8%). We found that older adults followed five distinct depressive symptom trajectories over 10 years. Mini-Mental State Examination scores, number of chronic diseases, educational level, gender, current employment, contact with children, and social activity were associated with a higher risk of these trajectories. Conclusions Depressive symptoms are associated with social networks as cognitive function scores increase and number of chronic diseases decrease. Interventions to strengthening existing social networks and developing relationships should be tailored to target specific needs for each trajectory, and chronic disease management, including cognitive function, may be beneficial in preventing depressive symptoms among older adults. KEYWORDS Older adults, Depressive symptom, Trajectory, Latent class growth analysis, Korean

